# Case Report: Synchronous aspirin-induced ileal ulcer and ascending colon adenocarcinoma: diagnostic challenges and pathophysiological insights from an incidental intraoperative finding

**DOI:** 10.3389/fmed.2025.1643706

**Published:** 2025-09-24

**Authors:** Zejin Zhao, Yue Xiao, Hui Zhao, Jian Li, Jinlong Liu

**Affiliations:** ^1^Department of Hepatobiliary Surgery, The Affiliated Hospital of Chengde Medical University, Chengde, Hebei, China; ^2^Department of Gastrointestinal Surgery II, Tangshan People’s Hospital, Tangshan, Hebei, China; ^3^Hebei Key Laboratory of Panvascular Diseases, Chengde, Hebei, China

**Keywords:** synchronous lesions, colon cancer, ileal ulcer, aspirin, mucosal damage

## Abstract

Synchronous gastrointestinal lesions are rare, especially when colon cancer is complicated by non-specific ileal ulcers. This article presents the case of a 70-year-old man who was admitted to the hospital with a colonic space-occupying lesion detected during the physical examination. An unexpected intraoperative finding revealed a synchronous ileal lesion with signs suggestive of malignancy. The patient had no history of inflammatory bowel disease and was on regular low-dose aspirin (100 mg/d) for a long time. Colonoscopy showed a mass in the ascending colon, and biopsy confirmed the diagnosis of moderately differentiated carcinoma. In addition to the ascending colon tumor, an ulcer with focal necrosis and enlarged lymph nodes was observed in the ileum approximately 40 cm from the Bauhin valve, which was suspected to be malignant or heterogeneous. To ensure complete removal of the lesion, a partial resection of the right hemicolon combined with the ileum was performed, followed by bowel reconstruction using a single anastomosis technique. The right upper colon and terminal ileum were resected as a whole, measuring approximately 60 cm in length. Postoperative pathology confirmed the diagnosis of colonic adenocarcinoma (pT3N0M0) and non-specific ulceration of the ileum, while ruling out Crohn’s disease, infection and other potential causes. The combination of the patient’s medical history and the absence of evidence of metastasis suggests that aspirin-associated mucosal injury and distant pro-inflammatory mechanisms related to the tumor may be synergistically pathogenic. The patient’s postoperative recovery was smooth, with no complications. This case emphasizes the importance of comprehensive intraoperative exploration, highlights the key role of multidisciplinary collaboration in differential diagnosis and surgical decision-making, and provides valuable insights for the individualized management of synchronous gastrointestinal lesions.

## 1 Introduction

Synchronous gastrointestinal lesions are primary lesions that are found concurrently in two or more different parts of the gastrointestinal tract during the same hospital stay or surgical procedure, with an overall incidence of approximately 2–5% in colorectal cancer patients (1). Among these lesions, synchronous multiple colon cancers and colon cancer with adenomas are more common. In contrast, the coexistence of colon cancer and small bowel lesions is rare, especially the combination of ascending colon cancer with non-specific ulceration of the ileum, which has been rarely reported in the literature and lacks a clearly defined pathogenesis (2, 3). Non-specific ileal ulcers represent a group of ulcerative diseases of the ileum that lacks specific pathological manifestations and a clear etiology. They present with a wide range of clinical manifestations and pathological findings, often characterized by linear or irregular ulcers that are superficial or extend deep into the submucosal layer. These ulcers may be related to factors such as drug use, infections, autoimmune reactions or ischemia (4). The diagnosis of isolated ulcers of the ileum is particularly challenging and needs to be differentiated from Crohn’s disease, intestinal tuberculosis, infectious enteritis, and drug-induced enteropathy (5, 6). Aspirin, an antiplatelet drug, is widely used in the secondary prevention of cardiovascular diseases, and a large number of studies have confirmed that its long-term use reduces the risk of colorectal adenomas and carcinomas (7). However, aspirin may also cause damage to the mucosa of the upper gastrointestinal tract and even the small intestine, inducing erosions, ulcers, and even bleeding, especially in the elderly or patients with other risk factors (6). Recent advancements in small bowel imaging techniques have led to the identification of more aspirin-associated lesions in the small bowel, although incidental intraoperative findings are still rare. This report describes the clinical features and management of an older male patient with colorectal cancer accompanied by a homogeneous ileal ulcer. The patient was admitted to Tangshan People’s Hospital in April 2025 following the detection of colonic lesions during a physical examination. With this case, we aim to enhance clinicians’ ability to recognize and respond to sudden combined lesions during surgery and to provide a practical reference for perioperative management of similar cases.

## 2 Case presentation

A 70-year-old man was admitted for evaluation of a mass in the ascending colon, which was detected during a routine physical examination. He had a history of coronary atherosclerotic heart disease, managed with long-term low-dose aspirin (100 mg/day) for over 5 years, without the use of other non-steroidal anti-inflammatory drugs, glucocorticoids, or immunosuppressants. He denied any prior gastrointestinal symptoms and had no history of inflammatory bowel disease, intestinal tuberculosis, or other chronic gastrointestinal disorders.

**Figure 1:**
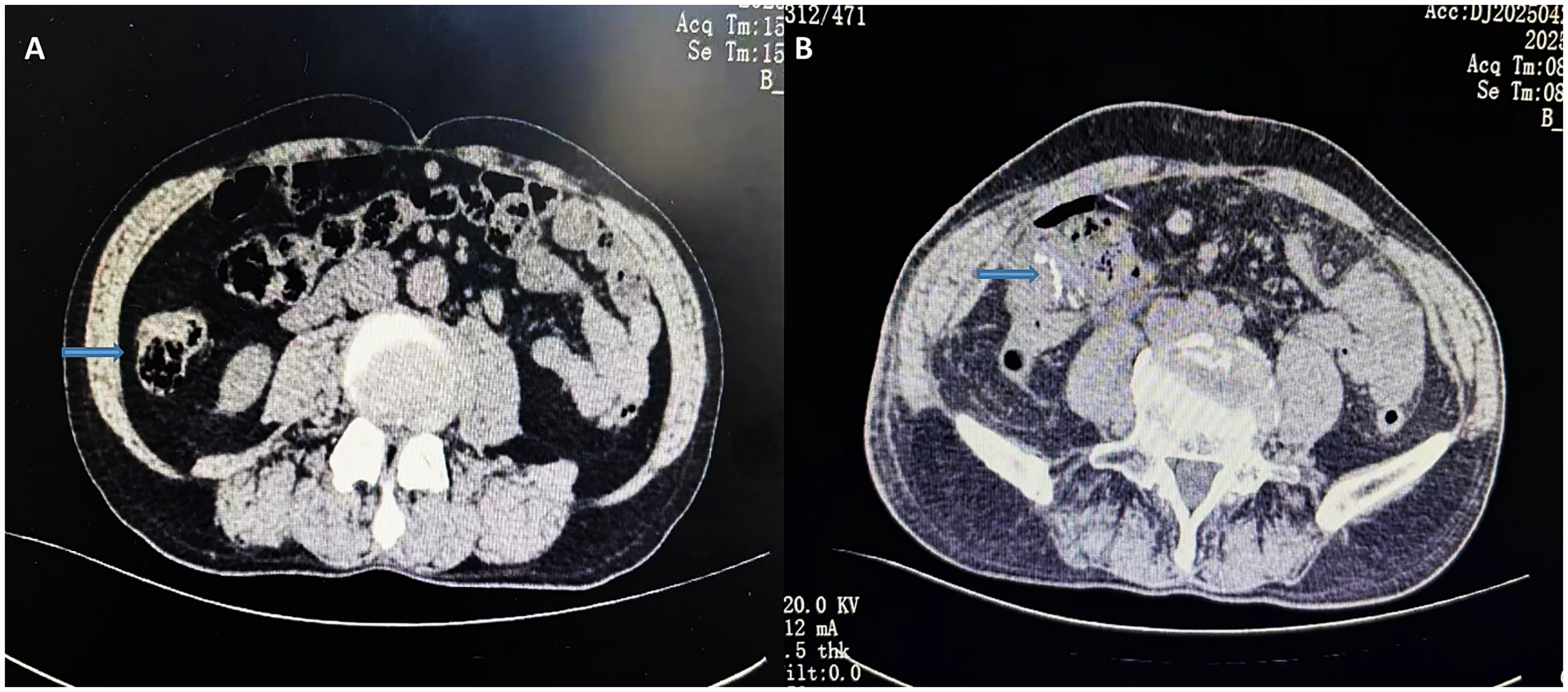
Preoperative CT transverse section of the abdomen **(A)**; postoperative CT transverse section of the abdomen **(B)**. The arrows indicate preoperative localized bowel wall thickening and the postoperative anastomosis.

**Figure 2:**
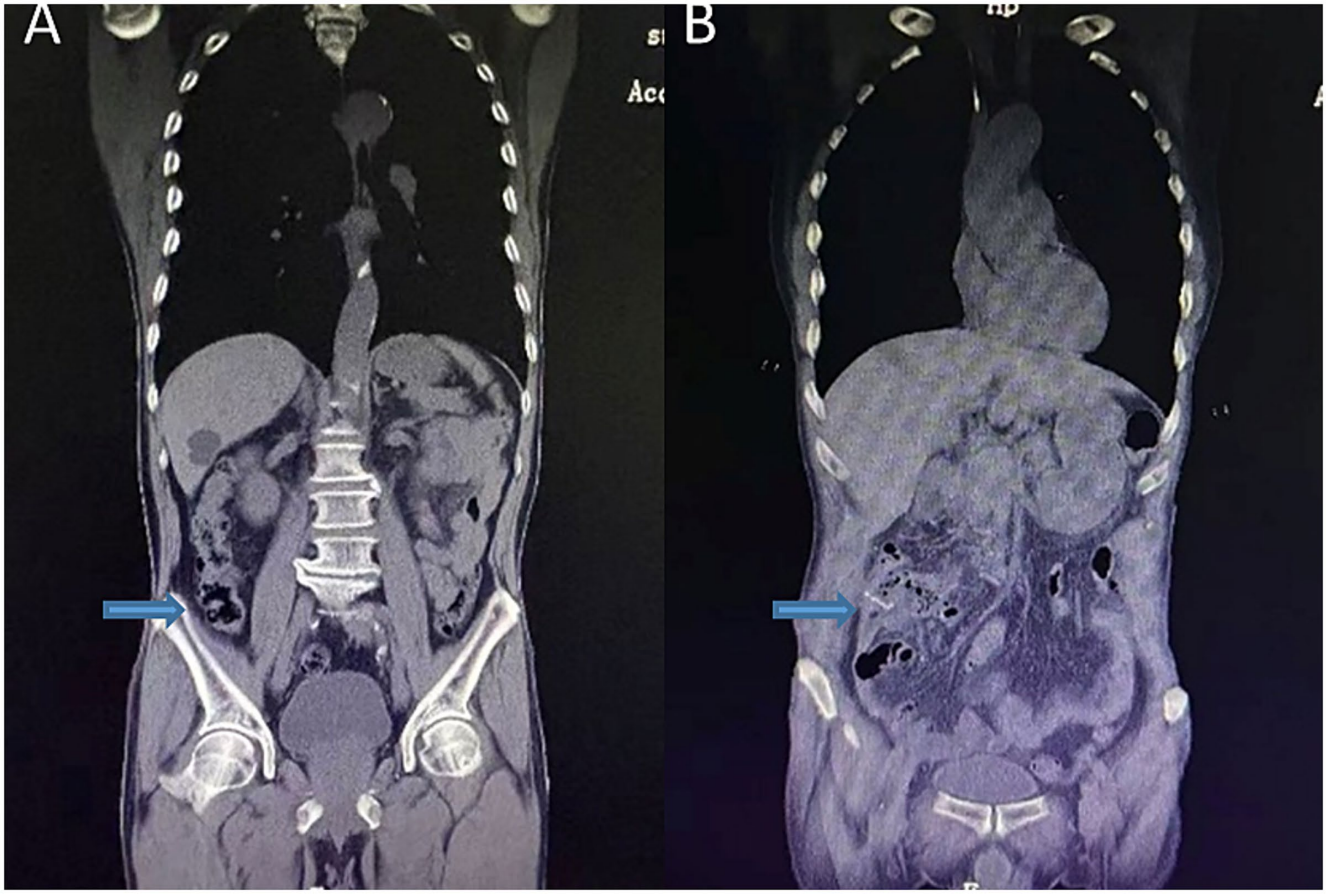
Preoperative abdominal coronal view **(A)**; postoperative abdominal CT coronal view **(B)**. The arrows indicate preoperative localized bowel wall thickening and the postoperative anastomosis.

Colonoscopy revealed a 3.0 × 2.5 cm elevated lesion in the ascending colon near the cecum, with surface erosion and contact bleeding. An additional adenomatous polyp (Yamada type IV) measuring 0.6 × 2.0 cm was identified in the sigmoid colon, approximately 15 cm from the anal verge. The biopsy of the ascending colon lesion confirmed moderately differentiated adenocarcinoma. Abdominal computed tomography (CT) demonstrated focal bowel wall thickening at the proximal ascending colon, with no evidence of regional lymphadenopathy or distant metastases (Figures 1A, 2A).

Following a multidisciplinary team discussion, a laparoscopic radical right hemicolectomy was planned. Intraoperative exploration revealed no ascites or peritoneal metastases. The primary tumor was resected en bloc along with the terminal ileum and surrounding mesentery. Incidentally, a 1.5 cm ulcerative lesion with focal necrosis and enlarged mesenteric lymph nodes was identified in the distal ileum, approximately 15 cm from the planned resection margin. Due to the uncertain malignant potential of the lesion, an additional 20 cm segment of the ileum was resected. Intestinal continuity was restored by performing an end-to-side ileotransverse anastomosis.

**Figure 3:**
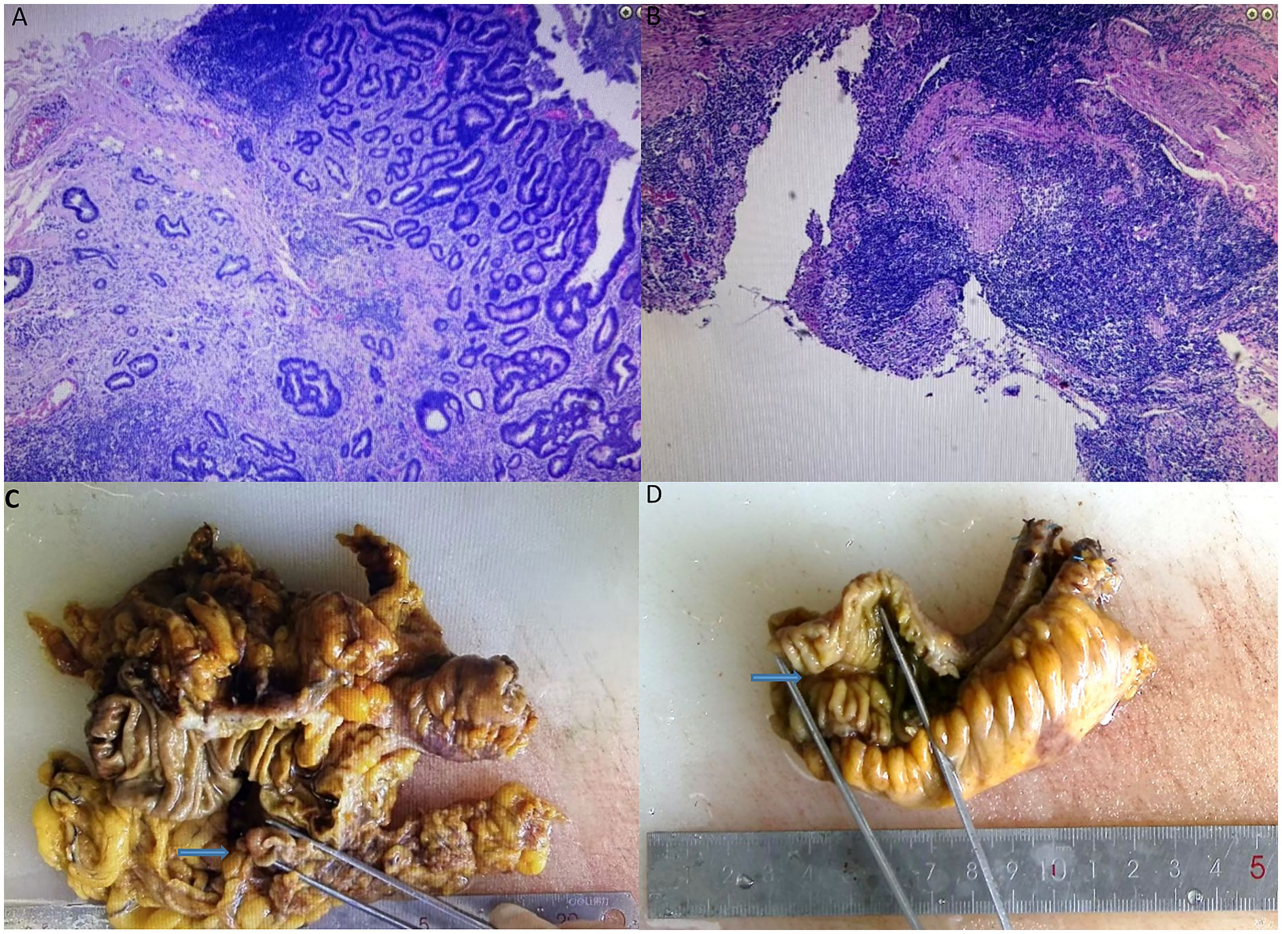
Postoperative immunohistochemistry of adenocarcinoma of the ascending colon **(A)** and non-specific ulcers of the ileum **(B)**. Postoperative pathology of adenocarcinoma of the ascending colon **(C)** and non-specific ulcers of the ileum **(D)**.

Histopathology confirmed moderately differentiated adenocarcinoma of the ascending colon infiltrating the subserosal layer, with negative margins and no lymph node metastases (pT3N0M0). The ileal lesion exhibited non-specific ulceration with mixed inflammatory cell infiltration, showing no evidence of Crohn’s disease, infection, or malignancy (Figure 3). Considering the patient’s history and pathological findings, aspirin-induced small bowel injury was deemed to be the most likely cause.

The postoperative recovery was uneventful. The patient resumed a semi-liquid diet on postoperative day 7, and follow-up imaging confirmed anastomotic patency (Figures 1B, 2B). He was discharged with recommendations to continue low-dose aspirin therapy in combination with mucosal protective agents, dietary optimization, and routine surveillance following the NCCN guidelines for stage IIA colon cancer.

## 3 Discussion

This report presents a case of a 70-year-old man diagnosed with adenocarcinoma of the ascending colon who was accidentally found to have a shaped storiform ulcer in the ileum, located approximately 40 cm from the ileocecal valve, during laparoscopic right hemicolectomy. The ulcer was confirmed as a non-specific mucosal ulcer by postoperative pathology. In this article, we discuss the possible pathogenesis of this case and analyze the identification, intraoperative management strategies, and differential diagnosis of synchronous small bowel lesions, aiming to enhance the understanding of these rare intraoperative emergencies and optimize the clinical decision-making process.

The patient had colon cancer with no obvious gastrointestinal symptoms. Intraoperatively, in addition to the primary tumor, a series of ulcers were found in the ileum far away from the site of the lesion, accompanied by focal necrosis and mild enlargement of mesenteric lymph nodes, suggesting the possibility of synchronous lesions. Due to the lack of rapid intraoperative pathological support, the surgeon assessed the morphology, location, and potential risk of malignancy of the lesion. Based on this evaluation, the decision was made to extend the resection with a combined resection of approximately 45 cm of the terminal ileum. The intestinal reconstruction was completed using a single-anastomosis telangiectomy technique. This intraoperative strategy reflects a prospective consideration of the risk of potentially multifocal tumors and the clinical decisiveness to achieve surgical “thoroughness, safety, and pathological follow-up” with limited information. Postoperative pathology confirmed moderately differentiated adenocarcinoma of the ascending colon with infiltration of the subplasma layer and negative lymph nodes (pT3N0M0), consistent with a diagnosis of colon cancer. The ileal lesions, on the other hand, showed non-specific ulcerative mucosal injury, with a mixed infiltrate of acute and chronic inflammatory cells seen microscopically, while typical inflammatory bowel disease features such as heterogeneous hyperplasia, granulomas, fissure ulcers and crypt fossa deformities were not found. Infectious enteritis and tumor metastasis were further excluded. Based on the patient’s medical history and pathological findings, aspirin-induced enteropathy was finally considered the most likely etiology.

Aspirin is a widely used antiplatelet agent and is frequently prescribed for the secondary prevention of cardiovascular disease. Although the mechanisms of gastric mucosal damage have been well studied, recent evidence suggests that low-dose aspirin (75–325 mg/day) can cause mucosal damage in the small intestine (6, 8). The mechanisms mainly include reduced synthesis of prostaglandin E2 (PGE2) due to COX-1 inhibition, disruption of intestinal barrier function, increased mucosal permeability, the release of local inflammatory factors, and the involvement of the Nrf2/Gpx4 pathway (9–11). Capsule endoscopy studies have shown that 10–20% of long-term low-dose aspirin users may develop small bowel erosions or ulcerative lesions, which are mostly asymptomatic or mild. However, a few cases have reported overt lesions that occurred in the presence of other coexisting inflammatory stimuli (12). The systemic inflammatory state induced by colon cancer may synergistically exacerbate small bowel injury through multiple mechanisms. Several studies have pointed out that gastrointestinal tumors can affect the barrier function and immune homeostasis of the distal intestinal mucosa through mechanisms such as the release of inflammatory factors (e.g., IL-6, TNF-*α*), signaling of exocytotic vesicles (extracellular vesicles), and activation of the Toll-like receptor pathway (13–17). In the context of long-term aspirin use, this synergistic inflammatory milieu can induce focal mucosal necrosis, ulceration, and even hemorrhage, providing a plausible explanation for the presence of isolated ileal ulcers observed in this case, which are located far from the tumor site.

In addition, this case also highlights the importance of clinical strategies for the management of incidental intraoperative lesions. The literature reports that the incidence of intraoperative detection of synchronous bowel lesions in colorectal cancer can be as high as 3–5%, including second primary cancers, polyps and inflammatory or infectious lesions (18). Although intraoperative freezing of pathology is ideal, in practice, individualized decision-making by the operator based on experience is often required due to limitations related to the lesion site, equipment conditions or pathological features. For lesions where malignancy cannot be definitively excluded, priority should, in principle, be given to achieving complete resection, obtaining definitive pathological staging and preserving adequate functional intestinal reserve. The unexpected detection of an ileal ulcer substantially influenced the surgical strategy, necessitating real-time adjustment of the resection extent to balance oncological safety with functional preservation. Leaving the lesion *in situ* carries the risk of overlooking a malignant or premalignant process, whereas extensive resection could increase the likelihood of postoperative nutritional and functional compromise. Therefore, intraoperative judgment must carefully weigh these competing factors, ideally supported by frozen section pathology when feasible.

Potential treatment options for incidental small bowel lesions include limited local excision, extended segmental resection, and intraoperative biopsy with deferred definitive surgery. Each treatment carries specific advantages and drawbacks: local excision maximizes bowel preservation but risks incomplete clearance, while extended resection ensures oncological safety but may impair long-term absorption. In this case, segmental resection was selected due to the uncertain malignant potential of the lesion, which was consistent with the principle of achieving oncological thoroughness while minimizing postoperative complications. The patient was ultimately diagnosed with stage IIA colon cancer (pT3N0M0), without lymph node metastasis. The postoperative course was uneventful, with no signs of anastomotic leakage, a smooth recovery of bowel function, and no complications, suggesting a favorable long-term prognosis.

It is of concern that small bowel injury is often clinically overlooked in long-term aspirin users. Routine screening for ileal ulcers in all elderly patients who use aspirin would involve an exceedingly large population and is therefore impractical. Instead, small bowel assessment may be considered selectively in high-risk patients with relevant symptoms or additional risk factors. Capsule endoscopy, small bowel microscopy and MR imaging of the small bowel are important tools for this purpose (19). Aspirin often leads to gastric ulcers (20), whereas small bowel ulcers are clinically rare and relatively difficult to diagnose due to the fact that the small bowel is not a routine site of examination and the limited use of small colonoscopy (21, 22). If aspirin-associated lesions are clearly identified, the use of prostaglandin E1 derivatives or proton pump inhibitors may also be considered to minimize intestinal toxicity, in addition to weighing the continuation of the drug (23). There is still a lack of in-depth research on the comorbidity of “aspirin-associated isolated ileal ulcer” and colorectal cancer, and this case provides clinical clues and empirical evidence for this research direction.

In summary, this case reflects the diagnostic and therapeutic challenges posed by the unexpected intraoperative discovery of a distal intestinal ulcer and highlights the following key points: high priority should be given to the identification and management of intraoperative synchronous lesions; aspirin-associated small bowel injuries are not uncommon in clinical practice and should be included in the differential scope of intraoperative and pathologic evaluations; wider resections and maintaining the anastomotic security are preferable in the absence of rapid pathological support; and, in the case of long-term users of antiplatelet agents, it is advisable to conduct systematic preoperative assessment of intestinal risks and to increase monitoring during postoperative follow-up. This case highlights the need to integrate multidisciplinary information to make accurate judgments in the context of multiple etiological factors. It emphasizes the need to develop the safest and most rational treatment pathway based on individual history and intraoperative findings.

## 4 Conclusion

In conclusion, the unexpected intraoperative discovery of a distal ileal ulcer in this case necessitated prompt adjustments to the surgical strategy, enabling complete resection and safe intestinal reconstruction. This case highlights the importance of intraoperative vigilance, personalized decision-making, and the consideration of medication-related enteropathy when managing incidental small bowel lesions during colorectal cancer surgery. This approach maximizes therapeutic efficacy and patient safety.

## Data Availability

The original contributions presented in the study are included in the article/supplementary material, further inquiries can be directed to the corresponding author.
